# Adoption Intention and Factors Influencing the Use of Gerontechnology in Chinese Community-Dwelling Older Adults: A Mixed-Methods Study

**DOI:** 10.3389/fpubh.2021.687048

**Published:** 2021-09-17

**Authors:** Huanhuan Huang, Zhiyu Chen, Songmei Cao, Mingzhao Xiao, Liling Xie, Qinghua Zhao

**Affiliations:** ^1^First Clinical College, Chongqing Medical University, Chongqing, China; ^2^Department of Nursing, The First Affiliated Hospital of Chongqing Medical University, Chongqing, China; ^3^Department of Orthopedic, The First Affiliated Hospital of Chongqing Medical University, Chongqing, China; ^4^Department of Urology, The First Affiliated Hospital of Chongqing Medical University, Chongqing, China; ^5^Department of Nursing, The First Branch of First Affiliated Hospital of Chongqing Medical University, Chongqing, China

**Keywords:** antioxidants, crop water productivity, irrigation regimes, Mediterranean region, nano-SiO2, principal components analysis, yield contributing traits

## Abstract

**Objective:** To explore the Chinese community-dwelling intention of older adults to adopt gerontechnology and its influencing factors.

**Design:** A mixed-methods sequential explanatory design with an inductive approach was employed. In phase 1, a self-made questionnaire was administered from August 2018 to December 2019. Multifactor logistic regression was used to analyze the adoption intention and factors influencing the use of gerontechnology. In phase 2, participants completed a semistructured interview to explore the adoption intention of a specific form of gerontechnology, *Smart Aged Care Platform*, from May to July 2020.

**Setting:** Twelve communities in three districts of Chongqing, China.

**Participants:** Community-dwelling older adults were included.

**Results:** A total of 1,180 older adults completed the quantitative study; two-thirds of them (68.7%) showed adoption intention toward gerontechnology. Nineteen participants (10 users and nine nonusers) completed the qualitative study and four themes were explored. Through a summarized understanding of the qualitative and quantitative data, a conceptual model of influencing factors, namely, predictive, enabling, and need factors, was constructed.

**Conclusions:** This study reveals that most Chinese community-dwelling older adults welcome the emergence of new technologies. However, there was a significant difference in the adoption intention of gerontechnology in Chinese community-dwelling older adults based on their sociodemographic and psychographic characteristics. Our findings extend previous technology acceptance models and theories and contribute to the existing resource base.

## Introduction

In 2019, the number of older people aged over 60 years in China reached 249 million, accounting for 17.9% of the total population (1). It is estimated that by 2050, the total number of older people will exceed 465 million, and 32.3% of them will be over 80 years old (2). As a result, China is not only the country with the highest proportion of elderly people in the world but also one of the countries with the fastest aging rate (3, 4). In addition to the one-child policy (5), population migration to cities (6) and an increase in the “empty nest” elderly population (7) have made it more difficult for Chinese modern families to maintain traditional home-based care (8). Furthermore, with the enhancement of living standards and lifestyles, the demand of older adults for old-age services has become varied; thus, personalized and smart services are being emphasized (9, 10). As a result, there is a growing awareness of the importance of developing and implementing comprehensive health solutions that are affordable, efficient, and of superior quality for older individuals (11).

Gerontechnology, a portmanteau of gerontology and technology and coined in Europe in the early 1990s, refers to techniques, technological products, services, and environments that are aimed at improving the daily life and aging of the elderly with technological advances (12, 13). Conceptually, gerontechnology can be divided into four categories according to its use (14): first, gathering continuous data (e.g., heart rate and motion) to monitor the performance of older adults or detect falls through wearable sensors (15); second, assisting older people cognitively and socially using, for instance, interactive robotic pets (16); third, providing care or monitoring of health from a distance, with the help of telecare or telemedicine (17); and finally, compensating for possible technology deficits in the home environment, which mainly refers to a smart home (18). Some gerontechnological solutions fulfill multiple purposes such as the Smart Aged Care Platform designed by our team (19). In this study, gerontechnology is used as an umbrella term for all the aforementioned technologies.

Gerontechnology helps older adults maintain their health and wellbeing in their homes (20), which is considered to be of positive significance to aging in place (21). Using telecommunications, gerontechnology can now provide health professionals and caregivers with remote access to older patients (22). In addition, gerontechnology has shown great potential in reducing escalating medical costs by eliminating the need for expensive and limited medical facilities (18, 23). In the context of the accelerated aging process, gerontechnology already has a broad market prospect (24), and delivering healthcare services based on gerontechnology has been one of the trends of providing care for older adults in China (25).

Compared to developed countries, the smart health technology of China is still in its infancy. It was only in 2012 that the Chinese Nation Working Commission on Aging first introduced the concept of gerontechnology (26). Thus, although several international studies have explored the perspectives of older adults on emerging technologies across the world (27–31), the data on China are still insufficient. Thus, this study aims to determine:

1. Chinese community-dwelling intention of older adults to adopt gerontechnology.
2. The factors influencing the adoption intention and use of gerontechnology.

## Methods

### Study Design

**Figure 1:**
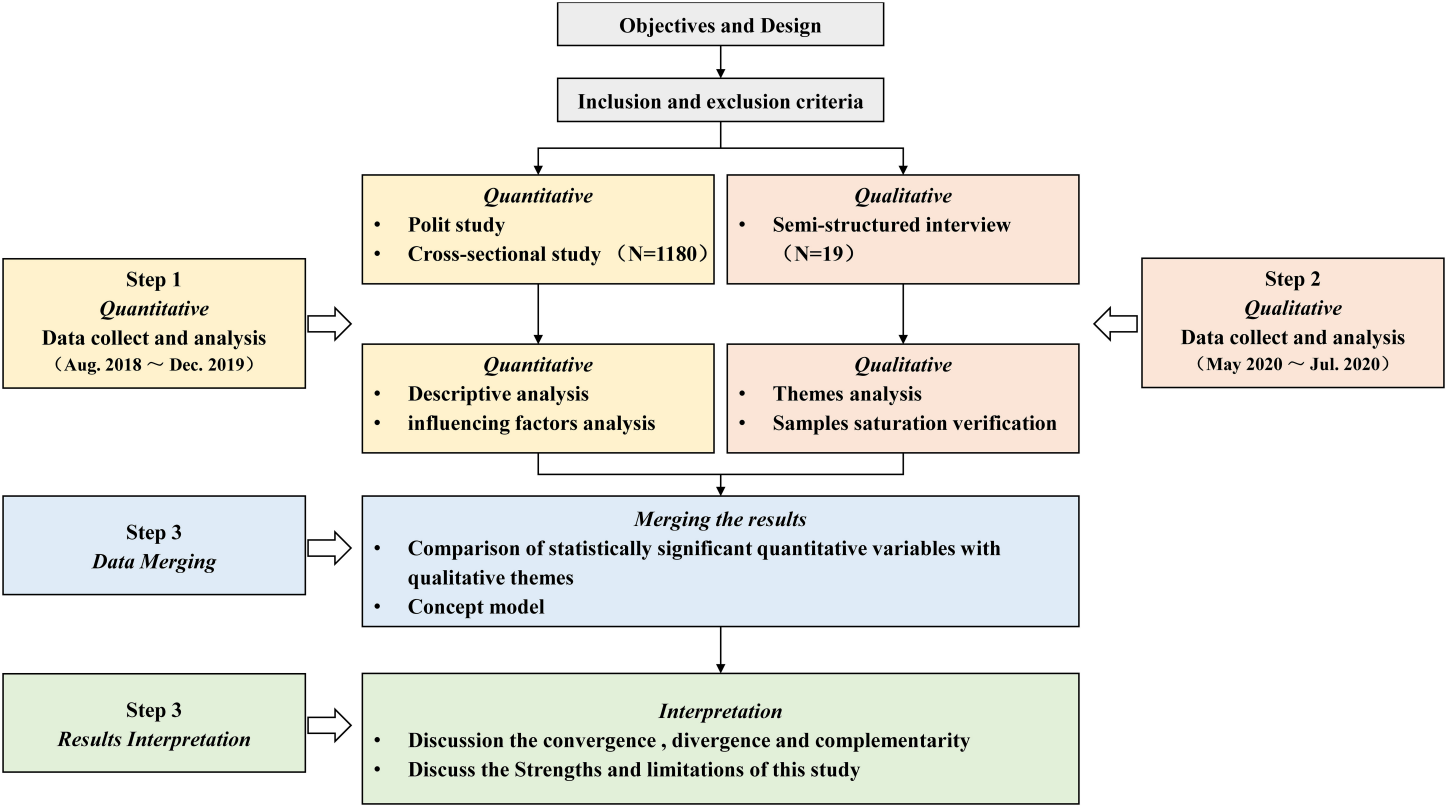
Flow diagram.

A mixed-methods sequential explanatory design with an inductive approach (19) was employed. To explain the quantitative results in more depth, qualitative data comprising the perspectives of participants were collected. Such a sequence of research design had been supported by past studies (32). In phase 1, 1,180 community-dwelling older adults completed a self-made questionnaire from August 2018 to December 2019. Multifactor logistic regression was used to analyze the adoption intention and factors influencing the use of gerontechnology. In phase 2, 19 participants were recruited, who completed a semistructured interview, to explore the adoption intention of a specific form of gerontechnology, Smart Aged Care Platform, from May to July 2020. The flow diagram is shown in Figure 1. This method has previously been used to investigate gerontechnology adoption by using specific forms of gerontechnology such as near-field communication (NFC)-enabled light systems (33) and soft service robots (34).

**Figure 2:**
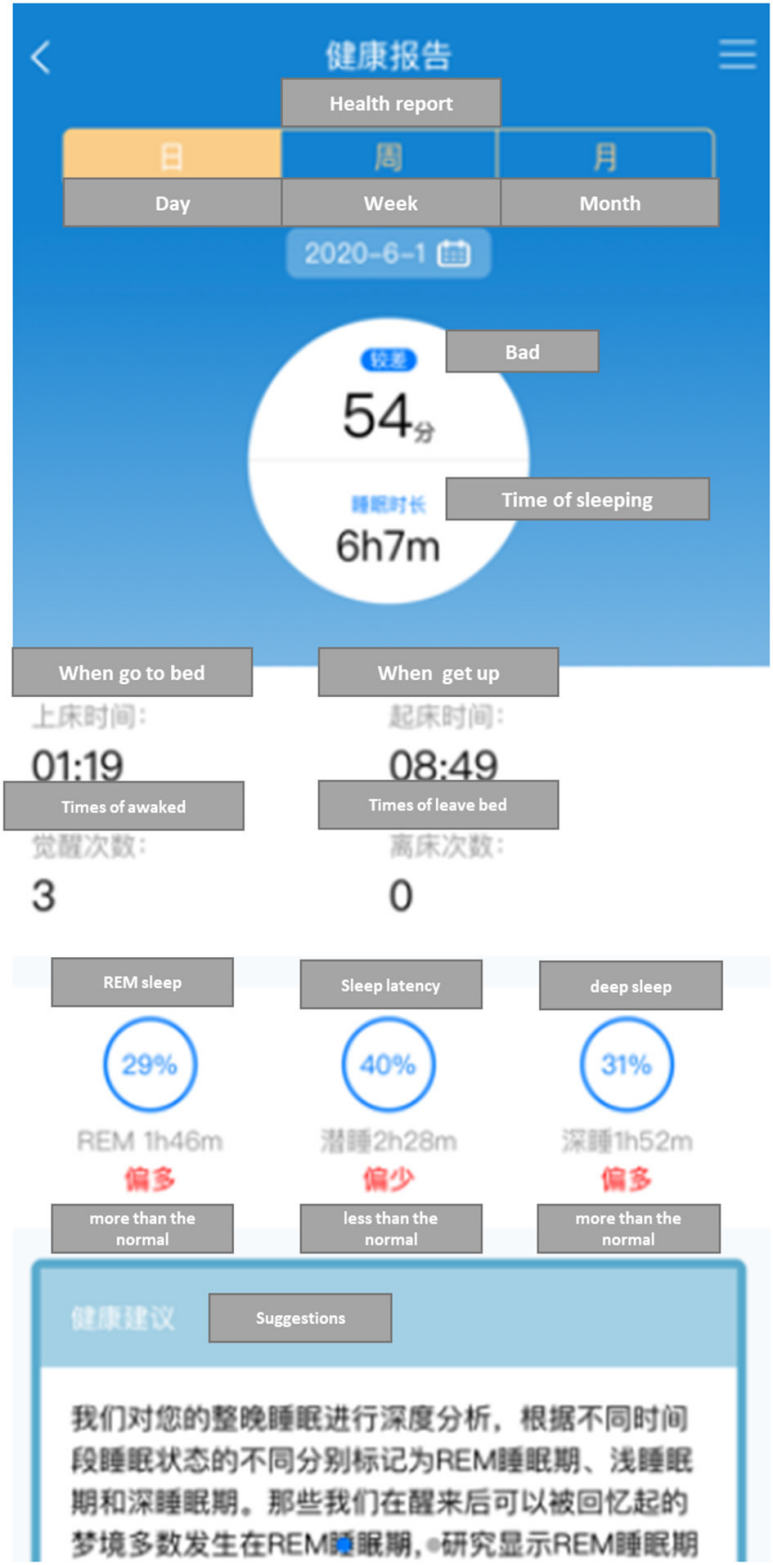
The sleep monitoring and reporting interface of Smart Aged Care Platform.

The Smart Aged Care Platform was designed by our team and empirically tested in Chongqing since January 2020 (35). Up to July 2020, 180 older adults were registered on the platform. The platform was developed to improve the way of life of older adults by helping them live independently. It was designed based on a hybrid aware model (23, 36), which consists of five subsystems: physiological data sensing system, environment data sensing system, location data sensing system, activities data sensing system, and decision support system. Table 1 summarizes the features and functions of each subsystem, and Figure 2 presents the sleep monitoring interface of the Smart Aged Care Platform.

**Table 1:** User portal (Smart Aged Care Platform) feature summary.

**Subsystem**	**Feature and function**
Physiological data sensing system	Continuous data of the human body (e.g., breathing, pulse, blood pressure, temperature) would be collected and reported by wearable devices and multi-modal biosensors.
Environment data sensing system	Environmental data (e.g., time, temperature, humidity, sound, light) would be monitored and reported by environmental sensors.
Location data sensing system	The real-time position would be reported and the action trajectory would be recorded.
Activities data sensing system	Activity log (e.g., sleeping, rest, exercise) would be monitored based on location and the time of stay.
Decision support system	Normal and abnormal activities would be classified, and professional suggestions would be provided according to the comprehensive health report.

### Setting

Chongqing, the largest city in the southwest, is one of the four major municipalities in China, and one of the most rapidly aging modern cities (37). In 2018, the number of older adults over the age of 60 in Chongqing reached 7.195 million, accounting for 21.13% of the total population. Thus, considering the economic level, the number of older people, and other comprehensive factors, 12 communities in three districts of Chongqing were selected as the main site for the study, as it is very probable that the findings would be similar throughout another metropolis of China.

### Participants

#### Quantitative Research

Convenience sampling was carried out in 12 communities in three districts of Chongqing from August 2018 to December 2019. The inclusion criteria were: (1) participants aged over 60 years, (2) those who have been living in the community for more than 6 months, (3) those who could correctly understand the contents of the questionnaire, and (4) those who do not have experience using gerontechnology. Participants who were diagnosed with critical or end-stage diseases, severe psychosis, or cognitive impairment were excluded. Based on the sample size estimation method for multivariate analysis study, the sample size should be 10–20 times the questionnaire variables (38). Hence, a sample size of approximately 260 participants was required. Finally, a total of 1,200 questionnaires were distributed, of which only 1,180 were received, with an effective recovery rate of 98.33%.

#### Qualitative Research

Data collection was carried out in the same communities as the first phase from May to July 2020. Due to the differences in e-health literacy and expression ability among different types of older people, the purposive sampling method was used to recruit participants to maximize the variation, in which education level, age, gender, experience using gerontechnology, and other factors were particularly considered. Subsequently, 19 participants were interviewed (*n* = 10 users; *n* = 9 nonusers). Users were those who had registered for the Smart Aged Care Platform and had logged in within the last 5–7 months. Nonusers were older adults who had neither registered for the platform nor used other kinds of gerontechnology.

### Tools

#### Quantitative Research

A self-made questionnaire was used, which consisted of three parts: (1) demographic information, namely, gender, age, education level, marital status, monthly income, and type of medical insurance; (2) health status information, namely, self-assessment of health level, chronic disease, and disability; and (3) adoption intention toward gerontechnology, which was assessed by asking participants if they “would intend to use or purchase gerontechnology-related services or products in 6 months” (0 = No; 1 = Yes).

#### Qualitative Research

A face-to-face, semistructured interview was conducted to explore and expand the potential factors that affect the adoption intention of gerontechnology by Chinese community-dwelling older adults. Participants were asked to discuss their perceptions about the rise of gerontechnology, any good or bad experiences they have had, the main challenges they face regarding adoption, and optimization advice or anticipation of their wants.

### Data Collection

#### Quantitative Research

The investigators are uniformly trained medical students, namely, five undergraduate and two graduate students, who were instructed to obtain the consent of the participants and their guardians before the investigation, and to inform all participants of the purpose and content of the study before it began. In the survey, a unified introduction was provided to guide participants through the process of answering questions. The filling of the questionnaire was standardized by the investigators according to the answers of the participants. After the end of the survey, data were collected, and their validity and completeness were checked.

#### Qualitative Research

The first and second authors are registered female nurses who have worked in geriatric nursing for a long time and have experience in conducting qualitative studies. They conducted nine and 10 interviews, respectively, and the duration of each interview was about 40–60 min. All the participants were informed of the objective and significance of this study before the interview. All the interviews were conducted as per the outline, and no specific guide was further required. All interview data were recorded and preserved after getting permission from all the interviewees.

### Data Analysis

#### Quantitative Research

All the data were verified and checked by the two authors in phase 1, and then imported into Stata version 14.0 (StataCorp LP, 2015) for statistical analysis. Frequency, constituent ratio, and mean ± standard deviation ($\bar{x}$ ± S) were used for descriptive analysis; *x*^2^ test was used for comparison; and multifactor logistic regression was used to analyze the influencing factors. The test level was bilateral test α = 0.05.

#### Qualitative Research

NVivo version 11.0 (QSR Inc., Melbourne, Australia, 2015) was used for data transcription. A qualitative thematic analysis was used for data analysis (39). The following steps were involved: First, at the end of each interview, the third author was mainly responsible for transcription, while the fourth author was responsible for checking its consistency and accuracy. The two did not participate in the interview sessions. Second, the researchers reviewed each transcript line by line to become completely familiar with the content. Then, all the data were initially coded and similar data were classified to form different topics (40). In addition, a list of themes was returned to the participants to reach a consensus on the validity again. Themes were defined and named until the final findings were negotiated among team members. Recruitment stopped when the data reached saturation, that is, when there were no new codes and topics obtained (41). To verify the saturation, five new participants were recruited. The results showed that the resulting topic contained all the important codes and topics put forward by the newly included participants, so it is considered that the samples at this stage are suitable (42).

### Participant and Public Involvement

All the participants were recruited in two parts by the local community health center through recruitment advertisements, and the location of the study was the generally quiet and undisturbed conference room of the agency. Participants and the public were not involved in the design of the quantitative research but were invited to assist in the confirmation process of the qualitative research.

### Ethics

Before the commencement of the study, approval was obtained from the ethics committee of the institution (approval number: 2019-105), and all the participants provided oral consent and willingness to complete the investigation. Transcripts and analysis records were stored on a secure cloud-based server. The anonymity of the respondents was protected in the form of digital codes.

## Results

### Demographic Results

A total of 1,180 participants were included in the quantitative research, whereas in the qualitative research, 19 older adults were invited to be interviewed. Table 2 provides the demographic information of all the participants.

**Table 2:** Demographic information of all the participants.

**Variable**	**Items**	**Phase 1** **(***N*** = 1,180, %)**	**Phase 2** **(***N*** = 19, %)**
Gender	Female	575 (48.73%)	9 (47.37%)
	Male	605 (51.27%)	10 (52.63%)
Age (year)	60–69	534 (45.25%)	13 (68.42%)
	70–79	373 (31.61%)	3 (15.79%)
	≥80	273 (23.14%)	3 (15.79%)
Living place	District	325 (27.54%)	3 (15.79%)
	Main urban	855 (72.46%)	16 (84.21%)
Education level	Illiterate	76 (6.44%)	2 (10.53%)
	Primary school	297 (25.17%)	1 (5.26%)
	Junior high school	427 (36.19%)	2 (10.53%)
	High school	221 (18.73%)	6 (31.58%)
	Above high school	159 (13.47%)	8 (42.11%)
Marital situation	Married	963 (81.61%)	13 (68.42%)
	Unmarried^a^	217 (18.39%)	6 (31.58%)
Monthly income (yuan)	<999	137 (11.61%)	2 (10.53%)
	1,000–2,999	381 (32.29%)	7 (36.84%)
	3,000–4,999	550 (46.61%)	9 (47.37%)
	>5,000	112 (9.49%)	1 (5.26%)
Type of medical insurance	New rural cooperative medical insurance	128 (10.85%)	7 (36.84%)
	Medical insurance for urban residents	281 (23.81%)	2 (10.53%)
	Medical insurance for urban workers	716 (60.68%)	10 (52.63%)
	Other^b^	55 (4.66%)	0 (0.00%)
Self-health assessment	Very poor	20 (1.69%)	0 (0.00%)
	Poor	115 (9.75%)	2 (10.53%)
	Fair	431 (36.53%)	5 (26.32%)
	Good	383 (32.46%)	10 (52.63%)
	Very good	231 (19.58%)	2 (10.53%)
Hypertension	No	638 (54.07%)	14 (73.68%)
	Yes	542 (45.93%)	5 (26.32%)
Diabetes	No	929 (78.73%)	10 (52.63%)
	Yes	251 (21.27%)	9 (47.37%)
Dyslipidemia	No	937 (79.41%)	17 (89.47%)
	Yes	243 (20.59%)	2 (10.53%)
Disability	No	1,123 (95.17%)	17 (89.47%)
	Yes	57 (4.83%)	2 (10.53%)

a *Unmarried includes single, divorced, and widowed*.

b *Other includes people with commercial health insurance or without any insurance*.

### Quantitative Results

A total of 811 (68.7%) older adults showed adoption intention toward gerontechnology. The multiple collinearity test among the variables shows that the variance inflation factor of the model is <10, so there are no multiple collinearities among the variables. Among them, the community older adults with different gender, living places, education levels, monthly income, type of medical insurance, self-health assessment, hypertension, diabetes, dyslipidemia, and disability had different intentions. The difference was statistically significant (*P* < 0.05), as shown in Table 3.

**Table 3:** Univariate analysis.

**Variable**	**Adopt** **(***N*** = 811)**	**x^2^**	***P***
Gender		3.461	0.043
Female	410		
Male	401		
Age (year)		0.595	0.743
60–69	373		
70–79	252		
≥ 80	186		
Living place		30.623	*P* < 0.01
District	184		
Main urban	627		
Education level		56.446	*P* < 0.01
Illiterate	38		
Primary school	180		
Junior high school	280		
High school	181		
Above high school	132		
Marital situation		0.991	0.32
Married	668		
Unmarried^a^	143		
Monthly income (yuan)		54.627	*P* < 0.01
<999	57		
1,000–2,999	269		
3,000–4,999	399		
>5,000	86		
Type of medical insurance		62.205	*P* < 0.01
New rural cooperative medical insurance	50		
Medical insurance for urban residents	191		
Medical insurance for urban workers	529		
Other^b^	41		
Self-health assessment		24.846	*P* < 0.01
Very poor	18		
Poor	77		
Fair	268		
Good	265		
Very good	183		
Hypertension		0.057	0.011
No	423		
Yes	388		
Diabetes		0.998	0.031
No	645		
Yes	166		
Dyslipidemia		2.924	*P* < 0.01
No	655		
Yes	156		
Disability		8.279	*P* < 0.01
No	762		
Yes	49		

a *Unmarried includes single, divorced, and widowed*.

b *Other includes people with commercial health insurance or without any insurance*.

The adoption of gerontechnology was set as a dependent variable, and the variables with statistical differences in univariate analysis were taken as independent variables. Multivariate logistic regression analysis was carried out to test the goodness of fit of the model, as shown in Table 4. Omnibus tests of model coefficients showed that *x*^2^ = 153.721 (*P* < 0.01), and the Hausman and Lemeshow test showed that *x*^2^ = 157.266 (*P* = 0.303 > 0.1). The results showed that the most significant factors for the community-dwelling older adults to adopt gerontechnology (*P* < 0.05) included above high school education (OR = 3.595, 95% CI = 1.707–7.571), monthly income between 1,000 and 2,999 yuan (OR=2.126, 95% CI=1.326–3.407), other type of health insurance (OR=3.336, 95% CI=1.573–7.072), poor self-health assessment (OR= 0.291, 95% CI= 1.061–1.376), hypertension (OR = 1.643, 95% CI = 1.216–2.221), diabetes (OR = 0.691, 95% CI = 0.497–0.96), dyslipidemia (OR = 0.567, 95% CI = 0.397–0.809), and disability (OR = 2.515, 95% CI = 1.118–5.658).

**Table 4:** Multivariate logistic regression analysis.

**Variables**	**Items**	**Control group**	**β**	**SE**	**OR**	**95% CI**
Gender	Female	Male	0.217	0.138	1.242	0.948–1.628
Living place	Main urban	District	0.246	0.162	1.279	0.931–1.757
Education level	Primary school	Illiterate	0.285	0.281	1.33	0.766–2.307
	Junior high school		0.355	0.291	1.427	0.807–2.522
	High school		1.164^**^	0.336	3.203	1.657–6.194
	Above high school		1.280^**^	0.38	3.595	1.707–7.571
Monthly income (yuan)	<2,999	<999	0.754^*^	0.241	2.126	1.326–3.407
	3,000–4,999		0.522^*^	0.267	1.685	1.998–2.844
	>5,000		0.338	0.372	1.403	0.676–2.909
Type of medical insurance	Medical insurance for urban residents	New rural cooperative medical insurance	0.784^*^	0.249	2.191	1.346–3.566
	Medical insurance for urban workers		0.768^*^	0.254	2.156	1.312–3.543
	Other^b^		1.205^*^	0.383	3.336	1.573–7.072
Self-health assessment	Poor	Very poor	−1.235	0.793	0.291	0.061–1.376
	Fair		−1.387^**^	0.775	0.25	1.055–1.141
	Good		−1.191	0.777	0.304	0.066–1.394
	Very good		−0.708	0.788	0.492	0.105–2.307
Hypertension	Yes	No	0.497^**^	0.154	1.643	1.216–2.221
Diabetes	Yes	No	−0.370^*^	0.168	0.691	0.497–0.96
Dyslipidemia	Yes	No	0.567^*^	0.181	0.567	0.397–0.809
Disability	Yes	No	0.922^*^	0.414	2.515	1.118–5.658

*^a^Unmarried includes single, divorced, and widowed*.

b *Other includes people with commercial health insurance or without any insurance*.

*SE, standard error; OR, odds ratio; CI, confidence interval*.

* *P < 0.05*;

** *P < 0.01*.

### Qualitative Results

Four themes and six subthemes were defined after thematic analysis.

#### Theme 1: Adoption Intention of Gerontechnology

The study found that most participants showed a positive attitude toward gerontechnology, believed that with the development of science and technology, it will be helpful to use emerging technologies to maintain and improve the quality of life in twilight years, and thought that this is a kind of care and well-being. “*The standard of living is getting higher and higher…. Even the elderly can enjoy the fruits of science and technology.”, Q4*.

Some participants reported that they had begun to use a variety of digital technologies at home, such as WeChat, Internet devices, and home sensors, to establish contact with their families and medical staff, and had had a pleasant experience. “*It's great that the machine can record the data directly and upload it to the cloud, then the doctor can track the changes. That's good.”, Q9*.

#### Theme 2: Social-Demographic Factors

##### Subtheme: Personality Traits

Some participants reported their confidence in the use of new technologies and products, and showed great curiosity; while others were relatively cautious, believing that the use of new technologies is time and energy consuming under the idea that traditional services can meet the demand. “*I am old in age, but young in mind. Although I am more than 70 years old, I always live with the mentality of learning.”, Q1. “I feel that my life is very comfortable now, and I don't want to put an extra burden on myself.”, Q13*.

##### Subtheme: Health Belief

Generally, most participants were worried about adverse events or complications of diseases. Those who had experienced such events before were more aware of the importance of health management in their daily life. “*Thirty percent depends on the doctor, and the rest depends on maintenance in your daily life.”, Q16*. One participant who once fell recalled that using home smart technology increased her sense of security and self-confidence to live alone at home. “*I fell and couldn't take care of myself for months. I'm afraid of falling again.…, technologies gave me a sense of security because I knew someone would come to help me in case of any emergency.”, Q7*.

#### Theme 3: Resource Factors

##### Subtheme: Product Characteristics

The sense of control over technology and products was the biggest concern of the participants. The older adults had observed the effects of aging on their daily life, which might have limited their ability to adopt new technology. “*I am worried that I cannot use it correctly…, after all, this is a high-tech creation.”, Q2*. The suitability of the technical features of the products affected the adoption intention of participants. “*If the screen is too small, it will make it more difficult for me to read…,” “if this product is a behemoth, I don't think most old people will like it.”, Q11*.

Reliability concerns whether the efficacy of the product or service felt by the user is stable and reliable. For example, some participants reported doubts about the accuracy of home technology measurement. “*I'm not sure if the data measured by these kinds of products are accurate, just like those in hospitals.”, Q5*.

##### Subtheme: Resource Accessibility

Participants also expressed concerns about the resources needed to use the product. The cost of technology was the biggest concern. “*Although there are many benefits, you know, there is very little money for the older adults.”, Q17*. Some participants suggested that national policies need to help and support older adults. “*If this is the future trend of our country, then the government should consider how to help the low-income elderly to bear these expenses.”, Q9*.

Some older people mentioned community and family resources and remarked that access to the Internet was a problem. “*If I live in the city, everything is fine, but if I go back to the countryside and I don't have Internet, then I have to abandon it.”, Q18*. Other participants had some doubts about the ability of the community to provide timely services. “*I mean, if we are far away, the healthcare workers in the ‘cloud’ will not be able to arrive at home to deal with the crisis as soon as possible.”, Q2*.

#### Theme 4: Need Factors

##### Subtheme: Assessed Needs

Assessed need refers to the actual poor physical condition of an individual. Many participants pointed out that if such technologies possess only conventional functions, such as measuring vital signs and environmental sensing, they would not be particularly attractive, but if the functions of these technologies and products are targeted to meet individual needs and can solve real problems in life, then they can be promoted for the well-being of the older adults. “*This is one of the doctor's suggestions, which allows me to avoid frequent hospitalization and reduce medical expenses.”, Q3*.

##### Subtheme: Perceived Needs

Perceived needs mean that the older population were not satisfied with their current health level or were willing to have a healthier experience; therefore, they were willing to try a helpful technology in achieving the same. Some participants pointed out that walking is a common leisure activity, and smart bracelets can help them understand and visualize their heart rate and evaluate their exercise intensity. Participants also pointed out that sleep quality is another important indicator of physical health. “*I often suffer from insomnia, and smart mattresses can be linked to my phone's application to help me understand how I sleep every night.”, Q1*.

### Emergence of the Qualitative and Quantitative Data

We found that the results of the two phases complemented each other. For example, income, health insurance policy, self-health assessment, and actual health status were considered as influencing factors in both quantitative and qualitative results. In addition, some other factors such as personal characteristics, resource accessibility, and product characteristics were supplemented and found in interviews.

**Figure 3:**
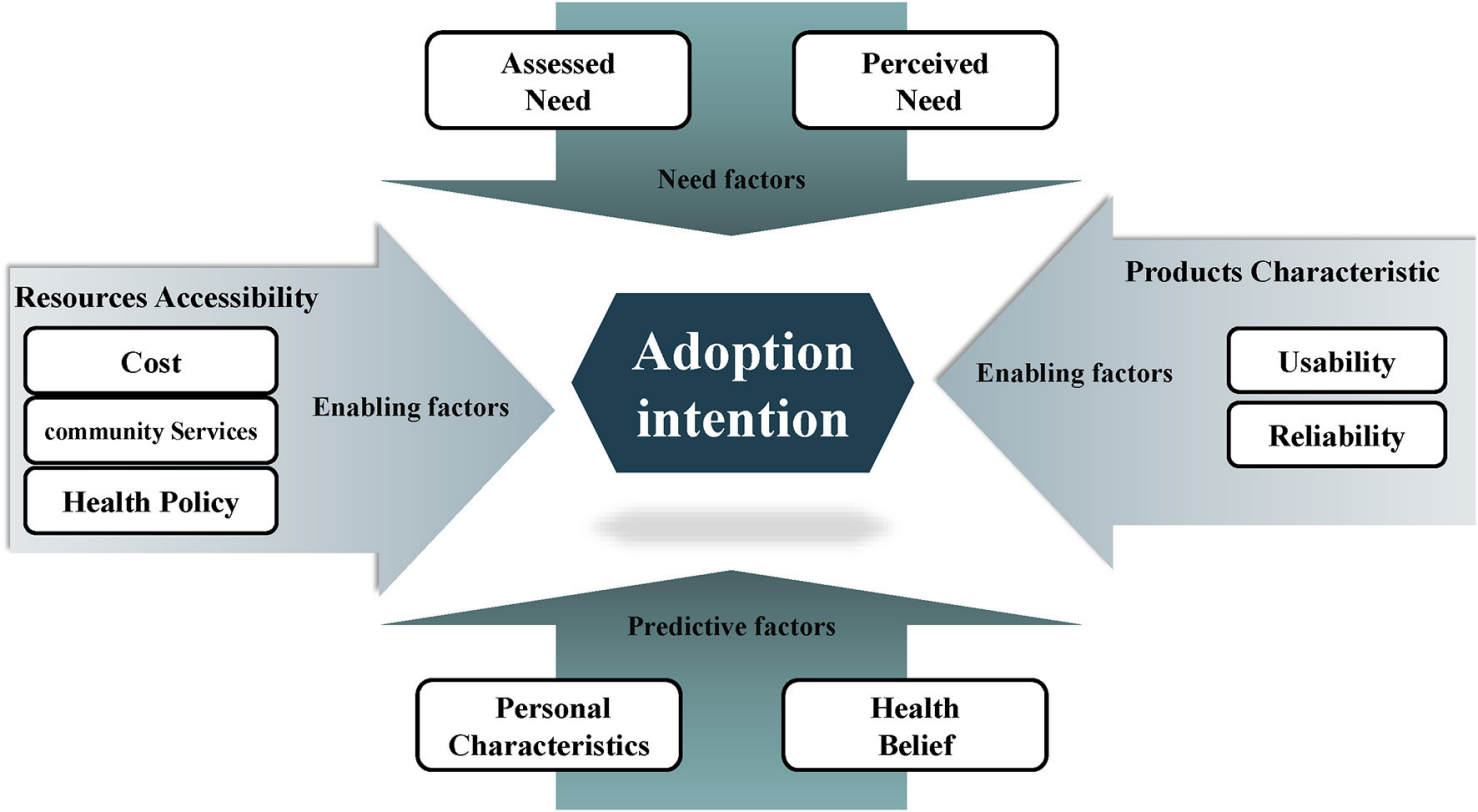
Conceptual model of influencing factors.

Therefore, we created a loose conceptual model based on the technology acceptance model (43) and health behavioral model of Anderson (44) to classify and visualize the factors found. As adoption intention is the main concern of this study, it is selected as the central action, which links the main results (Figure 3).

The definitions are determined as: (1) predictive factors, namely, personality traits and health beliefs, refer to the social-demographic characteristics that affect the adoption intention of older adults and use of gerontechnology. (2) Enabling factors, namely, product characteristics and resource accessibility, refer to the requirements of realizing technology utilization. (3) Need factors, namely, assessed and perceived needs, refer to the needs of community-dwelling older adults regarding the use of gerontechnology.

## Discussion

The results show that about two-thirds (68.7%) of the older population in this study shows adoption intention of gerontechnology, and welcomes the emergence of these technologies, which is consistent with the findings of many previous studies (45, 46).

However, we found a significant difference in the adoption intention of gerontechnology in Chinese community-dwelling older adults based on their sociodemographic characteristics. This result differs from the findings of a study conducted by Lim et al. (47), in which the subsequent impacts of usefulness evaluations on the intentions of older adults to use the NFC light system were explored. It was consequently found that older adults do not differ in their intentions to use gerontechnology when demographics and psychographics are considered. We feel that the results of our study differed because, in phase 1, the investigation was conducted with community-dwelling older adults, who do not have experience of using gerotechnology. Hence, their perceptions were based partly on their imagination and expectations regarding gerotechnology. According to existing literature, the evidence of the impact of demographic variables on the adoption of gerontechnology is inconsistent and weak (46, 48). Vroman et al. (31) proposed that age, education, and attitude are related to the adoption of technology. In addition to the above factors, de Veer et al. (46) found that men are more likely to adopt e-healthcare than women. Therefore, in the future, it is necessary to focus on the quantitative investigation of the impact of social-demographic characteristics on the adoption of technology.

More importantly, this study focused on the reasons, thoughts, and factors influencing the adoption intention of a specific gerontechnology from the perspective of community-dwelling older persons. The findings revealed that the factors influencing adoption intention are complex and systematic (49, 50), which involve predictive, enabling, and need factors. This knowledge can be applied to the design, improvement, optimization, and promotion of the technology industry, products, and services in the future.

### Predictive Factors

This study found that personality traits and health beliefs are important influencing factors and are deemed as predictive factors.

This study supports the evidence of the influence of personality traits on the adoption of technology. Qualitative research found that older users with optimistic characteristics and curiosity were more willing to try new products and technologies, which suggests that product developers should consider the psychological characteristics of the target consumers (49).

In addition, it was found that health beliefs were related to the behavior and willingness of older adults, which is consistent with previous research results (51–53). It is suggested that while formulating marketing strategies for geriatric technology, seminars must be designed simultaneously to raise awareness of its possible significant health benefits.

### Enabling Factors

Product characteristics are a significant part of enabling factors. One is usability, which mainly refers to the external features of technology, whose importance has been demonstrated in previous studies (51, 54, 55). Another is reliability, which emphasizes more on the inherent quality of technology, however, there are different views on this factor (45). Kaium et al. (56) found that system quality, expected performance, convenience, and social influence are of great significance to the continuous adoption intention of older adults in developing countries; however, service and information quality are not. While Hsieh et al. (57) found that system quality has the greatest impact on use intention and service quality has the strongest overall impact on user satisfaction (57). Overall, our study suggested that usability is as important as reliability; usability allows the participants to use the product correctly and reliability ensures that they can achieve the desired results. At present, more and more engineers and gerontologists are beginning to collaborate on issues at the intersection of technology, and listen extensively to the views of older users to find innovative solutions that are acceptable to older users (58).

In addition, we found that resource accessibility is also an important enabling factor, which consists of community services, cost, and health policy.

Our research results imply that the ability to obtain community services, such as medical care and the Internet, would influence the adoption intention of gerontechnology, which is consistent with related studies. Kohnke et al. (59) found that the support and assistance available during difficult times encourage the intention to use healthcare telemedicine equipment. This suggests that the design of health technologies for older persons should consider the obstacles that they may encounter in using the tool and carefully assess their current level of understanding.

This study suggests additional, potentially fruitful areas of inquiry. For example, our qualitative results found that older adults hope to ease economic pressure with the help of policies. A possible explanation is that since the long-term insurance system of China has not yet been established, most older people who lack a reliable and sustainable income are not willing to take the huge risk of losing money for owning these emerging technologies. Thus, this study suggests that bringing some gerontechnology into the reimbursement category of long-term care insurance in the future could be considered, to encourage older adults to stay at home longer and improve their quality of life with the help of technology.

Cost is examined in the research on almost all types of technologies and appears in various adoption models (51, 54, 55). According to Alsulami et al. (60), the cost of technology poses a great challenge to the behavioral intentions of older adults, in which the costs of installation, maintenance, and running are considered to be the main obstacles (61).

### Need Factors

This study shows that the technology adoption intention of the older population is mainly driven by demand, which can be the assessed need for real health situations and the perceived need for health promotion, which is similar to the results of a large number of previous studies (49). However, some studies believe that, compared with the two kinds of demand, the impact of perceived demand is less than that of assessed demand (51). Lee et al. (62) found that the adoption intention of information technology has nothing to do with self-assessed health. Therefore, it is necessary to further study the relationship between the adoption of communication technology and the physical and mental health of older adults in the future.

## Conclusions

This study used a mixed-methods approach to explore the complex factors that affect the adoption intention of older adults and constructed a conceptual model, comprising predictive, enabling, and need factors. This study provides theoretical insights on the adoption willingness of the older population in China and is expected to expand the previous technology acceptance models and theories, and to contribute to the existing knowledge database.

## Limitations

This study has four main limitations that could pave the way for further research. First, the relationships between the influencing factors in the conceptual model need to be verified statistically. Second, questionnaires measuring the intention to use gerontechnology have been developed by other scholars (63, 64) recently, and thus, it is suggested that these tools be translated into Mandarin to quantify and verify the intention to gerontechnology. Third, in phase 2, the data or description of the frequency, depth, and types of use of specific gerotechnology could be added to increase analytical depth. Finally, considering the close interaction among stakeholders, perspectives of informal caregivers toward gerontechnology are worth exploring.

## Data Availability Statement

The original contributions presented in the study are included in the article/supplementary material, further inquiries can be directed to the corresponding authors.

## Ethics Statement

The studies involving human participants were reviewed and approved by the hospital research ethics committee (approval number: 2019-105) and all the participants provided oral consent and willingness to complete the investigation.

## Author Contributions

QHZ, MZX, and LLX: conceptualization, validation, and supervision. HHH, SMC, and ZYC: methodology. HHH: software and writing original draft preparation. ZYC: formal analysis and visualization. HHH and SMC: investigation. SMC and ZYC: writing, reviewing, and editing. QHZ and MZX: funding acquisition. All authors have read and agreed to the published version of the manuscript.

## Funding

This research was funded by the Ministry of Science and Technology of China, Grant number 2020YFC2005900, Municipal Education Commission of Chongqing, China, Grant number KJCX2020018&yjg211006, and Science and Technology Committee of Chongqing, China, Grant number cstc2018jscx-maszdX00113. However, the funders had no role in the study design, data collection, management, analysis, or interpretation, manuscript writing, or the decision to submit the report for publication.

## Conflict of Interest

The authors declare that the research was conducted in the absence of any commercial or financial relationships that could be construed as a potential conflict of interest.

## Publisher's Note

All claims expressed in this article are solely those of the authors and do not necessarily represent those of their affiliated organizations, or those of the publisher, the editors and the reviewers. Any product that may be evaluated in this article, or claim that may be made by its manufacturer, is not guaranteed or endorsed by the publisher.
